# Epidemiological Overview of Colorectal Cancer in Kidney Transplant Recipients: A Systematic Review

**DOI:** 10.3390/cancers17203352

**Published:** 2025-10-17

**Authors:** Francesco Leonforte, Antonio Mistretta, Vito Nicosia, Maria Cristina Micalizzi, Davide Londrigo, Martina Maria Giambra, Giuseppe Roscitano, Pierfrancesco Veroux, Massimiliano Veroux

**Affiliations:** 1 Department of Integrated Hygiene, Organizational, and Service Activities (Structural Department), Health Management, University Hospital Polyclinic “G. Rodolico–San Marco”, 95130 Catania, Italy; leonfortefrancesco1@gmail.com (F.L.); davide.londrigo99@gmail.com (D.L.); 2 Department of Medical and Surgical Sciences and Advanced Technologies “G. F. Ingrassia”, University of Catania, 95131 Catania, Italy; anmistre@yahoo.it (A.M.); vitonicosia6@gmail.com (V.N.); 3 Helios Klinikum München West, 81242 Munich, Germany; mariacristina93mic@gmail.com; 4 Organ Transplant Unit, University Hospital of Catania, 95130 Catania, Italy; giambramartina@gmail.com (M.M.G.); giuseppe.roscitano@virgilio.it (G.R.); pveroux@unict.it (P.V.)

**Keywords:** colorectal cancer, kidney transplant, immunosuppressive therapy, solid organ transplant, epidemiology

## Abstract

**Simple Summary:**

Kidney transplantation improves both survival and quality of life, but it also requires long-term use of immunosuppressive medications. This increases the risk of several health problems, including cancer. Colorectal cancer is one of the most concerning conditions, yet its exact risk and characteristics in transplant recipients remain unclear. Our study reviewed the available evidence for the incidence of colorectal cancer in kidney transplant recipients, the risk factors and the clinical manifestations, and how it impacts on the post-transplant outcome compared with the general population. Transplant recipients may face a modestly higher risk, with cancers often detected at later stages and showing more aggressive features. The type of immune-suppressing treatment appears to play a key role. Overall, the evidence suggests that kidney transplant recipients may benefit from earlier and lifelong colonoscopy-based screening that is tailored to individual risk, with tailored immunosuppressive therapy to balance graft survival and cancer progression; strategies including earlier screening protocols may allow for earlier diagnosis and treatment, thereby improving the overall outcomes of kidney transplantation.

**Abstract:**

**Background:** Kidney transplant recipients (KTRs) experience improved survival and quality of life compared to dialysis treatment, but chronic immunosuppression may increase the risk of de novo post-transplant cancer. Colorectal cancer (CRC) is increasingly recognized in this population. This systematic review aims to synthetize contemporary evidence on CRC epidemiology, outcomes, and risk determinants among KTRs. **Methods:** A comprehensive search for observational and registry-based studies reporting CRC in adult KTRs was conducted on PubMed, Scopus, Web of Science, and ProQuest. The studies found were subsequently subjected to screening, data extraction, and the risk-of-bias appraisal process. Due to heterogeneity, findings were synthesized narratively. **Results:** Twenty-six studies encompassing 863,005 KTRs met inclusion criteria: 22 retrospective cohorts, 1 prospective cohort, 2 cross-sectional, and 1 case-control. Absolute CRC occurrence varies by geography, population, and follow-up. Reported risks ranged from no excess to modestly elevated standardized incidence ratios (SIRs): ~0.76–3.60 overall, with a right-sided colon predominance. Overall, higher mortality and worse prognosis were reported in kidney transplant recipients with colorectal cancer compared to the general population, as a result of later-stage diagnosis and more aggressive histologies. Consistent risk factors included older age, time since transplantation, diabetes and metabolic comorbidities, prior dialysis duration/graft failure, and smoking; the female sex showed higher relative CRC risk in some cohorts. The remarkable role of immunosuppression profiles was consistently highlighted: cyclosporine—azathioprine maintenance and alemtuzumab induction were associated with higher CRC risk in large registries, whereas tacrolimus—mycophenolate regimens showed lower risk signals and mTOR inhibitors suggested possible protective effects. **Conclusions:** Contemporary evidence suggests a modest, heterogenous increase in CRC risk among KTRs, a proximal (right-sided) predominance, and a tendency toward advanced-stage presentation with reduced survival. These findings justify the need to consider risk-tailored, lifelong surveillance strategies anchored in a full colonoscopy, with earlier initiation in younger or otherwise high-risk recipients, alongside careful optimization and periodic re-evaluation of immunosuppression. Prospective multicenter studies and cost-effectiveness analyses should refine screening thresholds and therapeutic strategies. PROSPERO ID: CRD420251071658.

## 1. Introduction

Kidney transplantation represents a cornerstone therapeutic intervention for patients with end-stage renal disease (ESRD), offering substantial improvements in both overall survival and quality of life compared to dialysis [1].

In recent decades, advances in surgical techniques, perioperative management, and immunosuppressive protocols have markedly enhanced short- and long-term outcomes, resulting in increased graft survival and reduced early mortality [2]. However, as transplant recipients live longer, long-term complications have become more prominent in clinical practice [3]. Among the most serious complications is the development of de novo malignancies, which constitute a leading cause of late morbidity and mortality among solid organ transplant recipients (SOTRs). It is widely recognized that they face a 2- to 4-fold higher risk of developing various types of cancer compared with immunocompetent individuals [4,5]. This elevated oncological risk is primarily attributable to the long-term use of immunosuppressive therapies, which are essential to prevent graft rejection, but simultaneously impair immune surveillance mechanisms, particularly T-cell-mediated immune responses, thereby facilitating the development and progression of malignancies [6,7]. Additional contributing mechanisms include genetic predisposition, lifestyle-related factors, and chronic inflammation [8]. Notably, the highest risks are associated with Kaposi sarcoma, non-melanoma skin cancers (NMSCs), post-transplant lymphoproliferative disorders (PTLDs), and anogenital cancers [9,10].

Within the spectrum of post-transplant malignancies (PTMs), colorectal cancer (CRC) has emerged as a malignancy of particular concern in kidney transplant recipients (KTRs) [11,12]. Although CRC is common in the general population, its incidence, presentation, and outcomes may show remarkable variability among SOTRs. Nevertheless, several robust epidemiological studies have consistently demonstrated an increased risk of CRC in KTRs compared to the general population [11,13,14,15,16,17]. This has led to a growing consensus on the importance of implementing lifelong surveillance programs, emphasizing preventative strategies, optimizing the selection and adjustment of immunosuppressive regimens, and fostering multidisciplinary collaboration among transplant specialists, oncologists, gastroenterologists, and primary care providers [4,18,19,20].

The heterogeneity of findings related to CRC risk reported in observational studies and registry-based analyses highlights the need for a comprehensive systematic review that is capable of consolidating the available evidence, providing a more accurate overview, and critically evaluating risk factors. Such an effort would yield valuable insights for both clinical practice and future research.

The primary objective of this systematic review is to synthesize epidemiological evidence on CRC among KTRs. Specifically, it aims to provide detailed insights into incidence, prevalence, mortality, five-year survival, and risk measures such as relative risk (RR), hazard ratio (HR), standardized incidence ratio (SIR), incidence rate ratio (IRR), and odds ratio (OR), as reported in recent studies. Furthermore, the review seeks to identify clinical, demographic, and therapeutic risk factors, with particular emphasis on immunosuppressive regimens.

## 2. Materials and Methods

### 2.1. Overview

This systematic review was conducted in accordance with the PRISMA guidelines (see Supplementary Materials section) [21]. The review protocol has been officially registered in the international PROSPERO database (ID: CRD420251071658).

### 2.2. Search Strategy and Study Selection

PubMed, Scopus, Web of Science, and ProQuest online databases were searched to generate potentially relevant articles through structured queries (full search strategy is available in Appendix A). Filters were applied to exclude irrelevant records, restrict results to the past ten years, and include only English-language publications. The final search was completed in June 2025.

### 2.3. Eligibility Criteria

Inclusion and exclusion criteria were defined a priori, according to the PEO-S framework:

- **Population/Problem (P)**

Studies reporting exclusively or partially on adult humans (≥18 years) who had undergone kidney transplantation.

- **Exposure/Condition (E)**

Clinically or instrumentally confirmed colorectal cancer.

- **Outcomes (O)**

Epidemiological measures (e.g., incidence, prevalence, mortality, 5-year survival), and risk indicators (e.g., RR, OR, HR, SIR).

- **Study designs (S)**

Observational studies (e.g., cross-sectional, cohort, case–control) and registry-based studies.

The following types of studies were excluded:

- Animal or in vitro studies.
- Reviews, editorials, commentaries, letters to the editor, study protocols without original data, abstracts, case reports, or case series.
- Studies not published in English.

### 2.4. Screening

**Figure 1:**
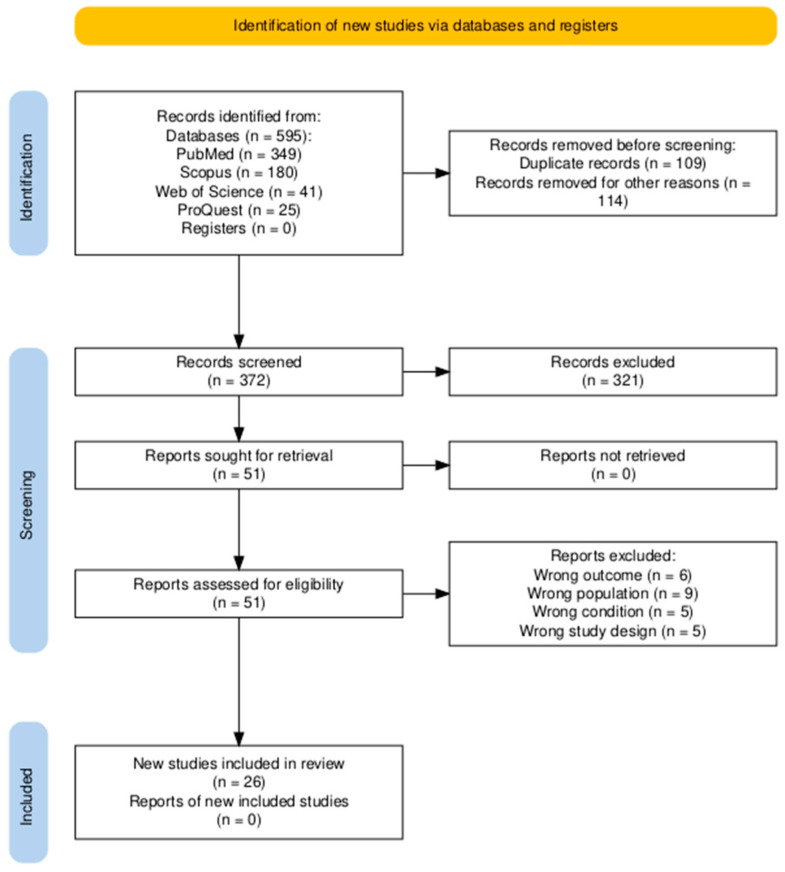
PRISMA flow diagram of the study selection process. A total of 595 records were identified through database searches, of which 223 were excluded as duplicates or for other reasons. After screening 372 records, 51 full-text reports were assessed for eligibility. Twenty-five reports were excluded due to wrong outcome, population, condition, or study design. Finally, 26 studies were included in the systematic review.

The study selection process was managed using the Rayyan© platform. Titles and abstracts were independently screened by two reviewers (V.N. and D.L.) in a blind manner. Any disagreements were resolved through a discussion with a third reviewer (F.L.). Subsequently, potentially eligible full-text articles were assessed following the same procedure. The PRISMA flow diagram summarizing the selection process is presented in Figure 1 [22].

### 2.5. Data Extraction and Synthesis

A standardized Microsoft^®^ Excel extraction form was purposely developed. The process was performed by two researchers (V.N. and D.L.) and verified by a third (A.M.). For each study included, the following information was collected where available: general details (author, year of publication, and country/setting), study design, sample size, key demographic and clinical characteristics, follow-up duration, immunosuppressive regimens and dosages, outcome data (incidence, prevalence, risk indicators, mortality rates, survival analysis, and risk factors), limits, conclusion, and implications.

Given studies’ heterogeneity, a qualitative synthesis was undertaken, integrating both narrative and tabular summaries in accordance with thematic analysis principles. The synthesis focused on primary and secondary outcomes, allowing for the identification of recurring patterns as well as remarkable discrepancies. Primary outcomes included the following: estimation of newly diagnosed CRC cases among KTRs expressed per 100,000 patient-years (incidence); overall proportion of KTRs diagnosed with CRC (prevalence); risk estimates (RR, OR, HR, SIR) compared with general or reference populations; general and disease-specific mortality rates obtained from follow-up studies; and medium-term prognostic analyses using Kaplan–Meier survival curves and 5-year survival estimates. Secondary outcomes included: role of clinical and demographic variables (e.g., age, sex, comorbidities); immunosuppressive therapeutic protocols affecting neoplastic risk and progression; and exploratory investigations.

### 2.6. Risk of Bias Assessment

The methodological quality of the included studies was assessed using tools appropriate to each study design. For cohort and case–control studies, the risk of bias was evaluated through the Newcastle–Ottawa Scale (NOS), a widely adopted tool for assessing the methodological quality of non-randomized observational studies. It evaluates studies across three domains: selection of study groups, comparability of cohorts, and ascertainment of exposure or outcome, awarding up to nine stars to reflect methodological robustness [23]. Concerning cross-sectional studies, risk of bias was assessed with the Joanna Briggs Institute (JBI) Critical Appraisal Checklist for Analytical Cross-Sectional Studies, which comprises eight criteria evaluating participant selection, measurement validity, confounding, and statistical analysis [24].

A quality appraisal was independently conducted by two researchers (V.N. and F.L.), with discrepancies resolved by a third (M.V.). Two graphical representations of the bias risk assessment were generated using the robvis tool [25].

## 3. Results

### 3.1. Overview

At the conclusion of the review process, a total of 26 studies published between 2014 and 2025 were included, encompassing 863,005 KTRs. Specifically, included studies were published in 2014 (n = 2), in 2015 (n = 4), in 2016 (n = 1), in 2017 (n = 2), in 2018 (n = 2), in 2019 (n = 4), in 2020 (n = 3), in 2021 (n = 5), in 2022 (n = 1), in 2023 (n = 1), and in 2025 (n = 1). The countries of origin of the included studies were primarily the United States (n = 5), South Korea (n = 5), Italy (n = 4), Taiwan (n = 2), Ireland (n = 2), and Spain (n = 2). Additional contributions came from Japan, Poland, Singapore, and Slovakia (one study each), while two studies were multinational.

The included methodologies comprised retrospective cohort studies (n = 22), prospective cohort studies (n = 1), cross-sectional studies (n = 2), and case–control studies (n = 1). The cumulative follow-up across the cohort studies showed considerable variability, ranging from 4 years to 41 years, with a mean duration of approximately 21.3 years. Sample sizes ranged from 63 to 272,325 participants per study. Further details of the included studies are presented in Table 1.

**Figure 2:**
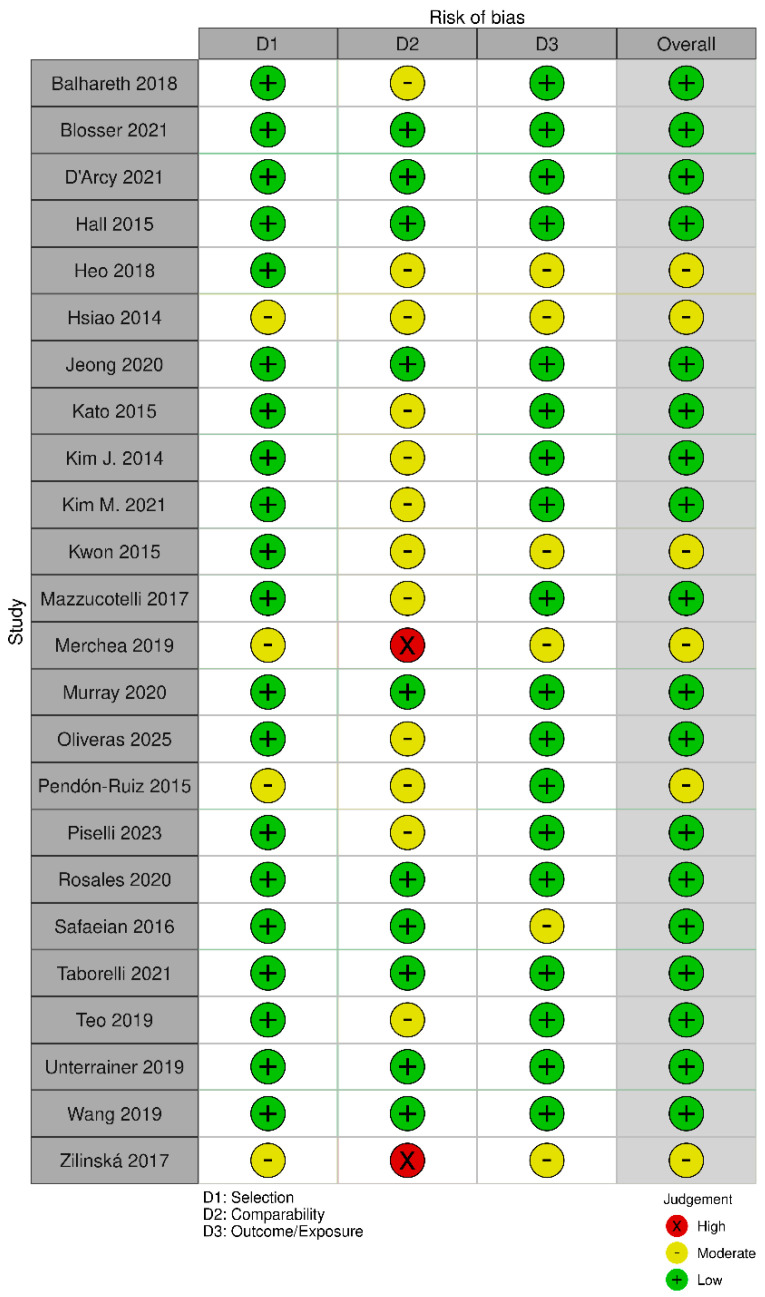
Risk of bias assessment for cohort and case–control studies ([26,27,28,29,30,31,32,33,34,35,36,37,38,39,40,41,42,43,44,45,46,47,48,49,50,51], see Table 1).

**Figure 3:**
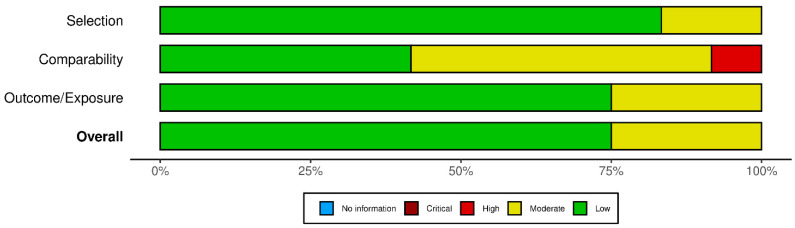
Bar plot for cohort and case–control studies.

**Figure 4:**
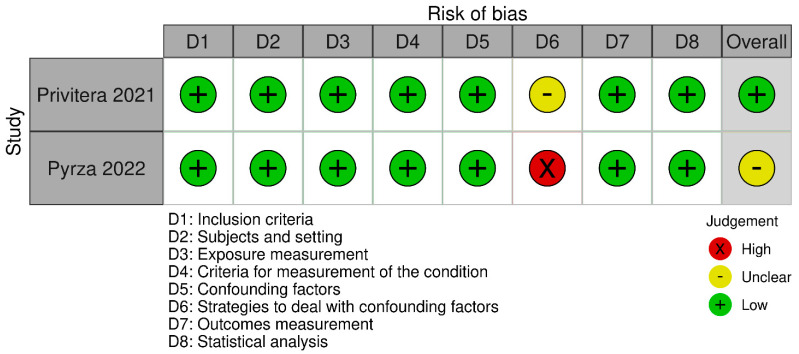
Risk of bias assessment for cross-sectional studies ([43,44]).

**Figure 5:**
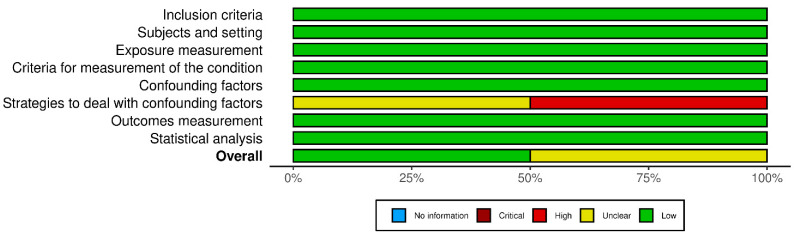
Bar plot for cross-sectional studies.

Among the 22 retrospective cohort studies, five were judged to have a moderate overall risk of bias, as was the single case–control study. The remaining 15 retrospective cohort studies, together with the sole prospective cohort study, were rated to be at low risk. The principal methodological concerns pertained to the comparability domain: particularly, the adequacy of adjustment for confounding factors. Within this domain, 12 studies showed a moderate risk of bias, while two exhibited high risk (Figure 2 and Figure 3). Of the two cross-sectional studies, one was deemed to have a low overall risk of bias and the other to have a moderate risk. In both instances, the main limitations were related to insufficient or absent strategies for addressing confounding (Figure 4 and Figure 5).

### 3.2. Incidence and Prevalence of Colorectal Cancer

Across the included studies, KTRs demonstrated a consistent incidence and prevalence of CRC, although absolute rates varied depending on population characteristics, geographical context, and follow-up duration. Balhareth et al. [26], analyzing Irish KTRs over a 37-year period, reported an overall CRC incidence of 697.5 per 100,000 person-year (0.6975%). Across decades, a temporal increase was evident, from 0.55% of transplants in the 1980s to 0.97% of transplants in more recent decades. Most malignancies (60.6%, n = 20) were in the right colon.

Blosser et al. [27], in a US registry cohort of 101,014 KTRs over 30 years, observed persistently elevated cancer incidence, specifically noting the absence of statistically significant temporal reductions in CRC risk, despite advancements in post-transplant care. Regarding rare CRC histotypes, D’Arcy et al. [28], analyzing a US national registry comprising 262,455 SOTRs (61.9% KTRs), documented an incidence of 2.23 per 100,000 person-year and an excess absolute risk (EAR) of 1.73 per 100,000 person-year for signet ring cell adenocarcinoma.

In Hsiao et al.’s study [31], CRC accounted for 5.6% of PTMs, ranking among the six most prevalent cancers. Jeong et al. [32] documented CRC as the second most common PTMS (11.2%) among 9915 South Korean patients. Likewise, Zilinska et al. [51] found CRC to be the third most common malignancy among KTRs (12,9% out of PTMs), following NMSCs and renal cell carcinoma (RCC). Kato et al. [33], studying a Japanese cohort, observed a PTMs incidence of 10.3%, with CRC comprising 6.5% of them (5/77), more often detected by symptom presentation (4/5) than routine screening (1/5). Similarly, Kim J. et al. [34], reported CRC representing 6.4% of PTMs in a study of 2365 KTRs. Kwon et al. [36] documented an 8.1% prevalence of advanced colorectal neoplasia in KTRs (including both advanced adenomas and cancers). Advanced adenomas (≥10 mm in diameter, >25% villous/tubulovillous histology and/or high-grade dysplasia) accounted for 6.5%, and CRC for 1.6%. Privitera et al. [43] identified 22 patients (13.7% of PTMs) with advanced colorectal neoplasia and 4 (2.5%) with CRC. Pendón-Ruiz de Mier et al. [41], in a Spanish cohort of 1450 KTRs, found CRC in 11% of PTMs (10/90). Murray et al. [39], in Ireland, observed CRC in 3.6% of KTRs diagnosed with malignancy (33/907). Taborelli et al. [47] documented 55 cases of CRC (46 in the colon, 2 at the rectosigmoid junction, 7 in the rectum) in an Italian cohort. Similarly, Piselli et al. [42] reported incidence rates increasing from 0.4 to 0.8 per 1000 person-years over 25 years. Mazzucotelli et al. [37], in 735 Italian KTR, described CRC in only five cases.

Pyrza et al. [44] reported an overall malignancy prevalence of 20% among KTRs, with CRC representing only 0.86% of cancers (3 cases out of 70 PTMs).

Finally, Teo et al. [48], in a Malaysian cohort of 491 KTRs, reported CRC in 9.7% of PTMs (3/31), contributing to an overall neoplasm incidence of 6.3%.

### 3.3. Risk for Developing Colorectal Cancer

In line with detected CRC cases, several studies reported risk measures. Blosser et al. [27] showed decreased but persistently elevated SIRs for overall malignancies, from 1.57 (1987–1996) to 1.28 (2007–2016), with CRC following similar trends. D’Arcy et al. [28] documented elevated SIRs for several rare malignancies, with colorectal signet-ring cell adenocarcinoma showing an SIR of 4.45 (95% CI: 3.02–6.31). Conversely, Heo et al. [30], in 1343 KTRs, reported an overall SIR for malignancies of 3.54 (95% CI: 2.89–4.29), with CRC contributing minimally. Jeong et al. [32] reported an overall SIR for CRC of 3.6 (95% CI: 3.0–4.3), while Mazzucotelli et al. [37] observed an SIR of 1.4 (95% CI: 0.4–3.2) and Kim J. et al. [34] reported an SIR of 1.0 (95% CI: 0.5–1.9), with no statistically significant risk increase in either genders compared to the general population. Piselli et al. [42] described a significantly lower CRC risk in KTRs than in the general population (SIR = 0.76; 95% CI: 0.58–0.99), with stable trends across periods (SIR = 0.61 in 1997–2004, SIR = 0.85 in 2005–2012, and SIR = 0.73 in 2013–2021). Adjusted IRRs also showed no significant temporal changes. Oliveras et al. [40], conversely, reported an SIR of 1.55 (95% CI: 1.28–1.85).

Kwon et al. [36] identified OR of 4.97 (95% CI: 2.18–11.29) for advanced adenomas, 6.25 (95% CI: 1.13–34.45) for CRC, and 5.37 (95% CI: 2.54–11.34) for advanced colorectal neoplasms in KTRs > 50 years compared to healthy controls. Unterrainer et al. [49], in a European multicenter cohort of 272,325 KTRs, found elevated HR for CRC recurrence among those with pre-transplant malignancies during the first 10 post-transplant years. Wang et al. [50], in Taiwan, observed an adjusted HR of 1.34 (95% CI: 1.11–1.62) for CRC in KTRs versus hemodialysis controls.

Privitera et al. [43] reported a non-significant increase in CRC risk (OR = 0.69), but a significantly increased risk of advanced adenomas (OR = 1.65), particularly in older patients.

Finally, Safaeian et al. [46], analyzing 224,098 SOTRs, documented 790 CRC cases, predominantly in the proximal colon. However, the risk in KTRs was not statistically significant (SIR = 0.99; 95% CI: 0.89–1.09).

### 3.4. Prognosis, Mortality, and Five-Year Survival

Two studies highlighted the aggressive presentation of CRC among KTRs. Balhareth et al. [26] reported that 87.8% of CRC cases were diagnosed at T3/T4 stages, with 45.5% showing nodal involvement and 42.4% exhibiting mucinous histology. Likewise, Merchea et al. [38] described 45.9% of CRC cases diagnosed at stage III/IV.

Mortality outcomes consistently indicated poorer prognoses among KTRs with CRC compared to non-transplant populations. Balhareth et al. [26] reported a mortality rate of 57.6% (19/33) at the time of analysis, and a five-year post-transplant survival rate of 87.9% (29/33). Malignancy was the direct cause of death in 13 cases, 7 of which were attributable to CRC. Blosser et al. [27] identified a 49% absolute risk of death with functioning graft (DWFG) within four years post-CRC diagnosis. Kim M. et al. [35] observed no statistically significant difference in recurrence-free survival (RFS) and overall survival (OS) between de novo and sporadic CRC, with five-year OS rates of 79.7% vs. 93.7%, respectively, when stratified by stage I and stage II/III disease. Jeong et al. [32] recorded 89 deaths among 598 patients with PTMs, of which 7.9% (n = 7) were due to CRC, yielding an overall standardized mortality ratio (SMR) for CRC of 2.7, which was higher among females (SMR = 1.8 vs. SMR = 1.1).

Merchea et al. [38] documented the median survival of 30.8 months, with five-year survival declining sharply by stage: 77% (stage I), 50% (stage II), 42% (stage III), and 0% (stage IV). Similarly, Pyrza et al. [44] and Zilinska et al. [51] both described high mortality among KTRs with CRC. Rosales et al. [45] reported 72 CRC-related deaths, corresponding to a SMR of 3.8 (95% CI: 3.0–4.8). Taborelli et al. [47] observed DWFG in 32.7% of CRC cases versus 11.8% in cancer-free controls, resulting in an HR of 3.51 (95% CI: 1.49–8.26). The HR for five-year survival was 1.17 (95% CI: 0.31–4.44).

Pendón-Ruiz de Mier et al. [41] noted markedly reduced mean survival among KTRs with solid organ cancer (2.09 years) compared to those with NMSCs (7.68 years). In Murray et al.’s study [39], the HR for CRC-specific mortality was 0.91 (95% CI: 0.58–1.43), showing no excess risk compared to the general population. Lastly, Privitera et al. [43] reported no recurrence at one year following advanced adenoma resections.

### 3.5. Risk Factors for Malignancies

The development of malignancies in KTRs was influenced by multiple risk factors, which were extensively reported throughout the reviewed literature. The most consistently recognized factor was immunosuppressive therapy [26,27,28,30,35,37,38,39,43,44,45,46,47,48,50,51]: specifically, cyclosporine use correlated with an elevated CRC risk [46,48]. Both the duration and cumulative load of immunosuppression played an incontrovertible role, with extended post-transplant duration significantly heightening risk [26,28,41,43,45,46]. Indeed, Safaeian et al. [46] noted a progressive increase in CRC risk, directly with time since transplantation (IRR = 1.86 for 8–12 years and IRR = 1.91 for >12 years, compared to <2 years). Similar findings were retrieved by Privitera et al. [43].

Recipient age was another widely recognized, non-modifiable risk factor. Older age was consistently associated with heightened neoplasm incidence [31,32,34,36,41,50]. Hsiao et al. [31] confirmed significantly elevated PTMs risk in recipients ≥ 65 years (HR = 5.32; 95% CI: 2.10–134.9) and 40–64 years (HR = 2.51; 95% CI: 1.11–5.66), compared with those < 40 years. Kwon et al. [36] reported a significant HR of 7.61 for advanced neoplasia in KTRs ≥ 50 years, whereas Kim J. et al. [34] found an HR of 1.05 (95% CI: 1.03–1.06) in those aged ≥ 40 years. Notably, several studies indicated earlier CRC onset in transplant recipients compared to the general population [26,35,43,46,50]. According to Safaeian et al. [46], proximal CRC incidence was higher in recipients at all ages, while distal and rectal CRC were elevated or equal to the general population in KTRs < 50 years, but reduced among those ≥50 years. Similarly, Wang et al. [50] displayed higher CRC risk among recipients aged under 50 years (adjusted HR = 2.08; 95% CI: 1.16–3.73), and Jeong et al. [32] showed a dramatic escalation in patients aged under 40 years (SIR = 40.0; 95% CI: 32.4–48.4).

Comorbidities such as ESRD, diabetes mellitus, obesity, coronary artery disease (CAD) and chronic inflammatory diseases also emerged as independent contributors to neoplastic risk [27,31,37,38,39,46,50]. Indeed, greater CRC susceptibility was highlighted by Safaeian et al. [46] among non-kidney SOTRs, particularly lung recipients with cystic fibrosis (IRR = 12.3; 95% CI: 6.94–21.9) and liver recipients with primary sclerosing cholangitis (PSC) or inflammatory bowel disease (IBD) (IRR = 5.32; 95% CI: 3.73–7.58), compared to kidney recipients, predominantly for proximal colon cancers. Oncogenic viral infections, although not directly linked to CRC, featured prominently as neoplastic risk factors in immunocompromised KTRs, particularly Epstein–Barr virus (EBV), human papillomavirus (HPV), cytomegalovirus (CMV) and JC Polyomavirus (JCV) [28,37,38,44,45,48,51].

Graft failure and dialysis duration prior to transplantation were additional determinants [30,32,37,44], with Jeong et al. [32] reporting an HR of 1.64. Furthermore, lifestyle-related factors such as tobacco smoking consistently identified as augmenting PTMs risk among transplant recipients [30,35,38,39,51].

Lastly, sex-specific differences were reported. Some studies observed higher relative PTM risk in females [30,35,47,50], with Heo et al. [30] documenting a higher SIR in women (SIR = 4,04 vs. SIR = 3,26), while Pendón-Ruiz de Mier et al. [41] and Teo et al. [48] described higher absolute risk in males. Wang et al. [50] highlighted that the CRC risk was significantly higher in female KTRs (adjusted HR = 1.48; 95% CI: 1.15–1.91). In the same vein, Kim M. et al. [35] and Jeong et al. [32] noted a significantly higher CRC risk in female KTRs (SIR = 2.54 vs. SIR = 1.67 and SIR = 6.0 vs. SIR = 2.3, respectively). Instead, Privitera et al. [43] found advanced adenomas more frequently in male recipients.

### 3.6. Immunosuppressive Regimen Analysis

The influence of immunosuppressive therapy on CRC incidence was explored in multiple studies. Hall et al. [29] reported an adjusted IRR of 2.46 (95% CI: 1.03–5.91) for CRC in KTRs receiving alemtuzumab induction therapy. Safaeian et al. [46] identified cyclosporine–azathioprine maintenance as a significant risk factor for proximal CRC (IRR = 1.53), whereas tacrolimus–mycophenolate regimens were associated with reduced CRC incidence (SIR = 0.79). Privitera et al. [43] suggested that prolonged immunosuppression may facilitate colorectal adenoma progression.

In the studies by Hsiao et al. [31] and Jeong et al. [32], no statistically significant association between immunosuppressive regimens and PTM risk was found, despite greater use of induction and maintenance therapies. Similarly, Privitera et al. [43] found no direct association between immunosuppressive type and CRC risk. In contrast, Kato et al. [33] observed that the use of calcineurin inhibitors (CNI) was significantly associated with increased PTM risk (adjusted HR = 1.77; 95% CI: 1.06–2.84, *p* < 0.001) compared to conventional therapy. Mazzucotelli et al. [37] linked induction therapy with elevated risk of Kaposi’s sarcoma (IRR = 3.52; 95% CI: 1.04–1.98) but not CRC. Likewise, according to Pendón-Ruiz de Mier et al.’s research [41], CNI therapy was specifically associated with a greater risk for developing NMSCs (OR = 2.17; *p* = 0.034). Teo et al. [48] identified cyclosporine use as an independent risk factor for malignancies, whereas induction agents, antimetabolites, corticosteroids, and the mammalian target of rapamycine inhibitors (mTORi) were not associated with higher PTM risk.

Several authors proposed a potential protective role of mTORi [33,35,45,47,51]. Kim M. et al. [35], replacing CNIs with mTORi in nine KTRs with de novo CRC, reported that eight remained recurrence-free, while one developed metastatic CRC. However, Unterrainer et al. [49] did not confirm consistent protective effects. Finally, Zilinska et al. [51] reported worse prognosis in patients not receiving induction therapy. Details of the immunosuppressive regimens predominantly used across the included studies are provided in Table 1.

## 4. Discussion

This systematic review provides an in-depth appraisal of colorectal cancer epidemiology among kidney transplant recipients, delineating a risk profile shaped by a complex interplay of factors [10,15]. Across studies, a consistently observed CRC burden is evident; however, heterogeneity in design, geography, calendar period, follow-up duration, outcome definitions, and reporting metrics (incidence versus prevalence; person-time rates versus proportions) limits comparability and inference. Small single-center series, often with very few CRC events, coexist with large registries, amplifying small-study effects and potential reporting bias. Apparent temporal increases may reflect enhanced detection (through improved diagnostics), longer observation, and evolving surveillance practices, rather than true risk escalation. The frequent absence of competing-risk methods further risks misestimating incidence in the presence of substantial non-cancer mortality. Overall, the literature suggests a modest but persistent post-transplant increase in CRC risk, which is broadly consistent with prior evidence, though effect sizes are heterogeneous and sometimes contradictory [15,52,53]. This variability reflects divergent comparators (general population, dialysis cohorts, or healthy controls), measures (SIR, HR, OR), and screening intensity. Reports of excess risk [32,36,40,50] coexist with null or sub-null SIRs [35,37,42,46], underscoring susceptibility to differential surveillance (greater colonoscopy use in higher-risk or symptomatic patients) and competing risks. Numerous potential confounders warrant explicit consideration: population size and age–sex composition; sociodemographic modifiers and lifestyle behaviors (e.g., smoking); and differences in healthcare access [54,55]. Additional determinants linked to generalized oncologic risk (underlying genetic predisposition; adverse comorbidity profiles such as diabetes, obesity, and chronic inflammatory conditions; and the composition, intensity, and cumulative exposure to immunosuppression) may compound baseline susceptibility and modify observed CRC associations. Several of these factors are modifiable, and support comprehensive, multidisciplinary post-transplant care that integrates lifestyle interventions and rigorous metabolic control into the routine follow-up [6,46,56]. Notably, the absolute risk of CRC remains lower than that of other post-transplant malignancies (PTMs) [4,56], among which non-melanoma skin cancer, Kaposi sarcoma, and post-transplant lymphoproliferative disorders are particularly characteristic, with well-established links to prolonged immunosuppression and oncogenic viruses such as Epstein–Barr virus (EBV) and human herpesvirus-8 (HHV-8) [4,10].

Across studies, KTRs with CRC more often present with advanced-stage disease and have poorer survival; however, effect estimates vary by study design, comparator, and endpoint (e.g., death with functioning graft, standardized mortality ratio, hazard ratio). Apparent excess mortality may partly reflect case-mix (older, more comorbid recipients), competing risks, and therapeutic limitations, while null findings in some cohorts highlight heterogeneity in surveillance and management. Stage-stratified analyses [57] reaffirm that prognosis remains strongly stage-dependent, rather than uniquely transplant-specific. The predominance of small single-center series and registries with limited adjustment raises concerns about selection and information bias. Collectively, the evidence supports heightened vigilance rather than a definitive conclusion of transplant-attributable lethality. Our synthesis indicates an association between CRC incidence/progression and immunosuppressive therapy, yet evidence comparing specific regimens is inconsistent and requires a context-sensitive interpretation that accounts for drug class, intensity, and cumulative duration of therapy. Some agents, including alemtuzumab and cyclosporine, have been linked to increased risk, whereas mTOR inhibitors may confer protection through antiproliferative and anti-angiogenic effects [29,58,59].

Mechanistically, immunosuppression, exacerbated by immunosenescence, diminishes antitumor surveillance and reprograms the tumor microenvironment toward immunosuppressive phenotypes, facilitating malignant clonal expansion. It also permits the reactivation or persistence of oncogenic viruses (e.g., EBV, HPV, HHV-8), promoting virus-driven cancers. In addition, agents such as cyclosporine and azathioprine may exert direct pro-oncogenic or genotoxic effects by impairing DNA repair pathways in immune cells [4,7,60].

However, the lack of standardized reporting of doses and treatment durations substantially weakens causal inference. Associations between particular immunosuppressants and CRC are highly sensitive to modeling choices; imprecision from small single-center studies and the possibility of selective reporting further limit confidence. Accordingly, regimen-specific risk estimates should be viewed as hypothesis-generating, rather than practice-changing. Further research is needed to define optimal surveillance intervals and therapeutic strategies, including the management of immunosuppression after a cancer diagnosis [61].

Histopathological data suggest a predilection for right-sided CRC, consistent with large registry studies [46,62]. This pattern aligns with evidence that right-sided colon cancers possess distinct molecular pathways and immune profiles compared with left-sided or rectal tumors, potentially heightening susceptibility to the effects of immunosuppression. In transplant recipients, underlying conditions such as cystic fibrosis and primary sclerosing cholangitis are associated with a higher risk of proximal colon cancer, likely through chronic inflammation and epithelial remodeling that predispose the individual to right-sided tumorigenesis [46,63]. Nevertheless, right-sided predominance may be influenced by differential colonoscopy practices. Regardless, colonoscopy should be favored over flexible sigmoidoscopy or fecal occult blood testing for screening in KTRs [64]. Colonoscopy visualizes the entire colon, detects proximal and distal lesions with greater yield for adenomas and advanced neoplasia, and permits immediate polypectomy. Compared with fecal occult blood testing, it has higher sensitivity and does not rely on repeated testing; moreover, immunosuppression-related bleeding may reduce the specificity of fecal occult blood tests, increasing false positives [65,66].

CRC in KTRs appears to display more aggressive clinical and biological features. Combined with later-stage diagnosis, this likely contributes to modestly worse outcomes, consistent with our survival analyses [67,68]. Although older recipients carry the greatest burden, several studies report a substantial proportion of cases in individuals younger than 50 years, plausibly reflecting tumor histology, prolonged immunosuppression, or diagnostic delays with a heightened impact in younger patients [68,69,70]. This occurs in a setting where guidelines and protocols often fail to actively offer a colonoscopy to KTRs under 50 years, despite their elevated risk [71,72]. To prevent missed or delayed diagnoses, our findings support earlier and more intensive surveillance, extending colonoscopic screening even to younger KTRs, and developing tailored, risk-stratified strategies that reflect the complexity highlighted in the prior literature [17,69].

To our knowledge, this is among the first systematic reviews to focus specifically on CRC epidemiology in KTRs with an emphasis on cancer-specific risk and mortality. Its principal strength is the large cumulative sample size, enabling exploration across diverse clinical scenarios and healthcare settings. Inclusion of registry-based and observational studies enhances the robustness of epidemiological observations. Nonetheless, several limitations merit emphasis. First, heterogeneity in patient populations, immunosuppressive protocols, and screening practices constrain comparability. Second, the predominance of retrospective observational designs and weak comparability domains in risk-of-bias assessments leave key associations under-adjusted and several conclusions plausibly artefactual, warranting a cautious, context-aware interpretation. Many cohorts conflate exposure windows, overlook therapy switches, and omit dosing/duration, introducing the risks of immortal-time bias and time-dependent confounding, despite statistical adjustment. Similar methodological constraints are well recognized in other areas of surgery and transplantation, where single-center retrospective studies with heterogeneous samples are common [73]. Because the evidence base is entirely observational, the review has a predominantly epidemiological slant; in the absence of randomized or prospective studies, generalizability is limited, and causality cannot be adjudicated. By contrast, patterns that persist in lower-bias strata (later stage at diagnosis and a shift toward proximal disease) are more defensible and should guide future research to optimize surveillance and clinical management. Third, social determinants of health (e.g., socioeconomic status, educational level, and health literacy) were rarely examined, despite their potential influence on outcomes. Finally, as with any systematic review, the possibility of having missed relevant studies cannot be excluded.

## 5. Conclusions

In summary, in this systematic review KTRs appear to have a modest heterogeneous increase in CRC risk. Recurrently observed patters are right-sided tumors, and later-stage diagnosis, with apparently poorer survival than in non-transplant population. These patterns seem to be influenced by age, cumulative exposure to immunosuppression over time since transplantation, metabolic comorbidities, and other additional risk factors. Signals from large cohorts and registries point to higher risk with cyclosporine–azathioprine maintenance and with alemtuzumab induction, lower risk with tacrolimus–mycophenolate, and a possible protective effect of mTOR inhibitors; however, exposure misclassification and time-dependent confounding limit causal inference. On this basis, it is suggested to consider risk-stratified, lifelong surveillance centered on a full colonoscopy, with earlier initiation for younger recipients or those carrying additional risk, integration within routine post-transplant care pathways, and periodic reassessment of immunosuppression when oncologic risk is a concern. To strengthen the evidence base, research should move beyond retrospective designs. First, prospective studies with a time-updated measurement of immunosuppressive exposure, standardized endpoints, and prespecified analyses are needed to clarify causal relationships between treatment patterns and cancer outcomes. Second, experimental and pragmatic trials should test whether initiating screening earlier, or optimizing intervals and modalities, improves stage at detection and survival in this population. Furthermore, large multicentric trials should evaluate tailored immunosuppression management (e.g., dose adjustments, class switches, or mTOR-based strategies) for their effects on both graft function and cancer risk. Third, focused pharmacoepidemiologic investigations are required to quantify neoplastic risk that is attributable to specific agents and combinations by using robust methods to address confounding and immortal-time bias. Additional priorities include cost-effectiveness analyses to guide policy, evaluation of equity and access (e.g., colonoscopy availability and health literacy) within surveillance programs, and translational studies using single-cell and other omics approaches to identify biomarkers that refine risk prediction and therapeutic tailoring. Embedding these elements within coordinated survivorship pathways, together with targeted modification of risk factors such as smoking and poor metabolic control, could facilitate earlier detection, reduce stage at diagnosis, and improve survival in this growing population.

## Figures and Tables

**Table 1:** Remarkable information from the included studies reporting colorectal cancer among kidney transplant recipients (n = 26). Data are presented as reported in the original articles. (Abbreviations: aIRR, adjusted incidence rate ratio; CI, confidence interval; CNIs, calcineurin inhibitors; CRC, colorectal cancer; HR, hazard ratio; IL2-RA, interleukin-2 receptor antagonist; IRR, incidence rate ratio; KTRs, kidney transplant recipients; mTORi, mammalian target of rapamycin inhibitors; OR, odds ratio; OS, overall survival; PTLDs, post-transplant lymphoproliferative disorders; PTMs, post-transplant malignancies; SIR, standardized incidence ratio; SOTRs, solid organ transplant recipients).

Author (Year)	Country	Study Design	Sample Size and Characteristics	Immunosuppressive Regimens and Dosages	Key Findings
Balhareth et al. (2018) **[26]**	Ireland	Retrospective cohort (1980–2017)	4731 KTRs; 33 developed CRC (20 male, 13 female); mean age at transplantation 51.5 years; mean time to CRC diagnosis 10.9 years	Immunosuppressive details not specified	Incidence: 697.5/100,000; CRC diagnosed 33/4731 (0.6975%); 60.6% were in right colon
Blosser et al. (2021) **[27]**	United States	Retrospective cohort (1987–2016)	101,014 KTRs (60.2% male, 39.8% female); cancer outcomes evaluated over 3 decades with 351,127 person-years of follow-up	Increasing use of polyclonal antibodies, tacrolimus, mycophenolate, IL-2R inhibitors; reduced maintenance corticosteroids over time	No significant trend changes in CRC incidence; IRR and SIR for CRC declined over time but not significant in adjusted analyses
D’Arcy et al. (2021) **[28]**	United States	Retrospective cohort (1987–2014)	262,455 SOTRs (61.9% KTRs); median age 48 years; median follow-up 139,3047 person-years	48% received induction therapy, details unspecified	SIR for signet ring cell adenocarcinoma of colon: 4.45 (95% CI: 3.02–6.31)
Hall et al. (2015) **[29]**	United States	Retrospective cohort (1987–2009)	111,857 KTRs; median follow-up 3.5 years	Muromonab-CD3, alemtuzumab, polyclonal antibodies, IL2R antagonists	Alemtuzumab associated with increased CRC risk (aIRR = 2.46); other regimens not associated with CRC significantly
Heo et al. (2018) **[30]**	South Korea	Retrospective cohort (2010–2014)	1343 KTRs (64.9% male, 35.1% female); mean age 48.5; 7.7% developed malignancy	Azathioprine, cyclosporine, prednisolone, mycophenolate mofetil, tacrolimus, and sirolimus	CRC incidence data not directly provided: colon (n = 1), rectum (n = 2). Non-statistically significant SIR for colon and rectum malignancies
Hsiao et al. (2014) **[31]**	Taiwan	Retrospective cohort (2000–2010)	642 KTRs; mean age 54.5 years (with cancer) or 42.4 years (without cancer); ~50% male and ~50% female; median follow-up 46.2 months; 8.4% developed cancers	Cyclosporine, Tacrolimus, Mycophenolate	CRC accounted for 5.6% of cancers (3/54 cases), incidence not reported in absolute terms
Jeong et al. (2020) **[32]**	South Korea	Retrospective cohort (2003–2016)	9915 KTRs (60.2% male and 39.8% female); median follow-up 4.87 years; median age at diagnosis 52.0 years; 6.0% PTMs	Use of basiliximab and anti-thymocyte globulin for induction; CNIs, mycophenolate, steroids for maintenance	CRC was 11.2% of post-transplant cancers (67/598); overall SIR for CRC = 3.6; SIR in male = 2.3; SIR in female = 6.0; SIR in <40y = 40.0; cancer-specific mortality for CRC = 7.9%
Kato et al. (2015) **[33]**	Japan	Retrospective cohort (1972–2013)	750 KTRs (454 male, 256 female); mean age at transplantation 38.9 years; 77 PTMs (10.3%); mean interval to cancer 134.5 months	CNIs, mycophenolate, prednisolone, antilymphocyte globulin or basiliximab for induction	CRC detected in 6.5% (5/77 cases, 4 in males and 1 in females), difficult to detect via routine screening methods
Kim J. et al. (2014) **[34]**	South Korea	Retrospective cohort (1989–2009)	2365 KTRs (61% male, 39% female); mean age at transplantation 39.4 years; 140 cancers (5.7%); mean follow-up 9.8 years	Predominantly basiliximab, daclizumab, OKT3, or antithymocyte globulin for induction; calcineurin inhibitors, corticosteroids, and either mycophenolic acid or azathioprine for maintenance	CRC accounted for ~6.4% of cancers (9/140, 4 in males, 5 in females); overall SIR of CRC = 1.0 (95% CI: 0.5–1.9), SIR in males = 0.6 (95% CI: 0.2–1.6), SIR in females (95% CI: 0.5–4.2)
Kim M. et al. (2021) **[35]**	South Korea	Retrospective cohort (2005–2016)	4264 KTRs; 66 de novo CRC; median follow-up 5.8 years; mean age at transplantation: 44.1 years; compared to matched sporadic CRC patients	CNIs, mycophenolate, and prednisolone	SIR for de novo CRC: 1.67 (95% CI: 0.98–2.64) in men, 2.54 (95% CI: 1.21–4.68) in women in KT recipients; survival like sporadic CRC; more colon than rectal cancer (*p* = 0.041)
Kwon et al. (2015) **[36]**	South Korea	Case–control (1996–2008)	248 KTRs with colonoscopy (155 male, 93 female); mean age at transplantation 52.6; mean follow-up 67.7 months; 900 age/sex-matched controls	Cyclosporine- or tacrolimus-based triple therapy, mycophenolate, azathioprine, mTORi	Advanced colonic neoplasms 8.1% vs. 3.7% in controls; CRC prevalence 1.6% (4/248); OR for advanced neoplasms 2.305 (95% CI: 1.29–4.09); stronger in >50y (OR 5.37)
Mazzucotelli et al. (2017) **[37]**	Italy	Retrospective cohort (1997–2012)	735 KTRs; follow-up 4858 patients-years	CNIs; monoclonal or polyclonal antibodies against T-cell antigens combined with conventional immunosuppressive drugs for induction	CRC reported in 5 cases (3 in males, 2 in females) among solid cancers; SIR = 1.4 (95% CI: 0.4–3.2)
Merchea et al. (2019) **[38]**	USA	Retrospective cohort (1987–2016)	63 patients with CRC after solid organ transplant (55.6% male, 44.4% female); majority kidney (44.4%); mean age at transplantation 57.3 years; median time to CRC 59.3 months	Immunosuppressive regimen not detailed	24.6% diagnosed at stage IV; 5-year OS 42.5%; right colon predilection (60.9%); poor survival in advanced stages. Precise incidence in KTRs unspecified
Murray et al. (2020) **[39]**	Ireland	Retrospective cohort (1994–2014)	3267 KTRs; 907 with cancer	Immunosuppression details not reported	CRC in 33 KTRs (3.6%), 18 in males and 15 in females. No increased risk of mortality in KTRs vs. general population for CRC; HR for all-cause mortality of 0.91 (95% CI: 0.58–1.43) and HR for cancer-specific mortality of 0.91 (95% CI: 0.58–1.43) for CRC
Oliveras et al. (2025) **[40]**	Spain	Retrospective cohort (2003–2021)	8037 KTRs (64.7% male, 35.3% female); median age at transplantation 57 years; median time to cancer diagnosis 5 years. 2013 PTMs	Polyclonal antibodies, CNIs, mTORi during the first 6 weeks post-transplant	122 CRC cases; SIR = 1.55 (95% CI: 1.28–1.85)
Pendon-Ruiz de Mier et al. (2015) **[41]**	Spain	Retrospective cohort (1979–2015)	1450 KTRs; 90 developed PTMs; mean age at transplantation 59 years (with cancer), 53 years (without cancer)	Triple therapy with CNIs, mycophenolate/azathioprine, and prednisolone. Induction with basiliximab or thymoglobulin	CRC in 11% of SOC (≈10/90 cases) CRC ~0.7% prevalence (3/194); survival after SOC diagnosis ~2 years
Piselli et al. (2023) **[42]**	Italy	Cohort (1997–2021)	11,418 KTRs (63.8% male, 36.2% female); median age at transplantation 50 years; 1646 PTMSs	1997–2004: predominantly cyclosporine 2005–2012: predominantly tacrolimus 2013–2021: predominant combinations including mTORi	CRC incidence: 0.4 per 1000 person-years in 1997–2004 (7 cases), 0.8 per 1000 person-years in 2005–2012 (29 cases), and 2013–2021 (23 cases). Adjusted IRRs = 1.54; 95% CI: 0.67–3.54 for 2005–2012 and IRR = 1.39; 95% CI: 0.59–3.29 for 2013–2021. SIR = 0.76; 95% CI: 0.58–0.99
Privitera et al. (2021) **[43]**	Italy	Cross-sectional (matched case–control)	160 KTRs vs. 594 controls; median colonoscopy after 6.4 years post-transplant	Tacrolimus, mycophenolate, steroids, cyclosporine, and everolimus. Three-drug regimens, with or without induction therapy	22/160 (13.7%) with advanced colorectal neoplasia, 4/160 (2.5%) with CRC; no increased CRC risk vs. controls (OR = 0.69); higher advanced adenoma risk (OR = 1.65)
Pyrza et al. (2022) **[44]**	Poland	Cross-sectional	350 KTRs; mean age 48 years; malignancies in 70 patients (20%)	CNIs, azathioprine, prednisone	CRC in 3 cases (0.86%); skin and PTLDs most common; limited CRC-specific data
Rosales et al. (2020) **[45]**	Australia and New Zealand	Retrospective cohort (1980–2016)	17,628 KTRs (61% male, 39% female); median age at transplantation 45 years; 1061 cancer deaths	CNIs, mTORi	Not mentioned
Safaeian et al. (2016) **[46]**	USA	Retrospective cohort (1987–2010)	224,098 SOTRs (58.2% kidney); 61.2% male and 38.8% female; median age at transplantation 48 years	Cyclosporine + azathioprine, tacrolimus + mycophenolate, induction therapy, others	790 CRC cases, overall SIR = 1.12; SIR = 1.69 for proximal colon cancer; SIR = 0.93 for distal colon cancer; SIR = 0.64 for rectal cancer; higher risk in liver and lung recipients (SIR = 2.34; SIR = 1.34, respectively), while non-significant in kidney recipients (SIR = 0.99; 95% CI: 0.89–1.09)
Taborelli et al. (2021) **[47]**	Italy	Retrospective cohort (1997–2017)	1425 KTRs with cancer vs. 2850 matched controls, 4275 individuals in total; 70 CRC cases	Regimens not fully detailed; mTORi associated with better survival after cancer	CRC in 55 cases (46 in colon, 2 in rectosigmoid junction, 7 in rectum)
Teo et al. (2019) **[48]**	Singapore	Retrospective cohort (2000–2011)	489 KTRs; mean age 47.1 years; median age at cancer diagnosis 50 years, median time to malignancy 2.6 years; 31 malignancies (6.3%) overall	CNIs, mycophenolate, azathioprine, corticosteroids. mTORi, CNIs + azathioprine, CNIs + mycophenolate	3/31 CRC cases (9.7%)
Unterrainer et al. (2019) **[49]**	Multi-national	Retrospective cohort (1984–2016)	272,325 KTRs (Collaborative Transplant Study database), of which 4184 had pre-transplant malignancies	CNIs, mycophenolate, azathioprine, steroids, prophylactic antibody induction therapy (IL2-RA, rATG, other)	No site-specific data for CRC; elevated incidence of CRC recurrence after transplantation (HR = 6.0; 95% CI: 2.7–13.5)
Wang et al. (2019) **[50]**	Taiwan	Retrospective cohort (2000–2008)	3739 KTRs (59.03% female, 40.97% male) vs. 42,324 dialysis patients; mean age KTRs 61.8 years.	Not specified	CRC incidence 372.9 per 100,000 person-years vs. 232.5 in non-KT; adjusted HR 1.34 (95% CI: 1.11–1.62), higher risk in women and <50 years
Zilinska et al. (2017) **[51]**	Slovakia	Retrospective cohort (2007–2015)	1421 KTRs; 85 malignancies (6%); median time to cancer 45 months, mean age at diagnosis 54 years	Tacrolimus, cyclosporine A, mTOR inhibitor (alone or with CNIs), mycophenolate, corticosteroids; IL2-RA (Basiliximab/Daclizumab) for induction or rATG for induction	11/85 were CRC (12.9%)

## Data Availability

A template reporting the extracted data may be available upon motivated request and approval by the research team.

## Supplementary Materials

The following supporting information can be downloaded at: https://www.mdpi.com/article/10.3390/cancers17203352/s1, Supplementary File S1: PRISMA checklist.

## Abbreviations

The following abbreviations are used in this manuscript:

CAD	Coronary artery disease
CMV	Cytomegalovirus
CNI	Calcineurin inhibitor
CRC	Colorectal cancer
DWFG	Death with functioning graft
EAR	Excess absolute risk
EBV	Epstein–Barr virus
ESRD	End-stage renal disease
HPV	Human papillomavirus
HR	Hazard ratio
IBD	Inflammatory bowel disease
IL2-RA	Interleukin-2 receptor antagonist
IRR	Incidence rate ratio
JCV	JC Polyomavirus
KTR	Kidney transplant recipient
mTORi	Mammalian target of rapamycine inhibitors
NMSC	Non-melanoma skin cancer
OR	Odds ratio
OS	Overall survival
PSC	Primary sclerosing cholangitis
PTLD	Post-transplant lymphoproliferative disorder
PTM	Post-transplant malignancy
rATG	Rabbit Anti-Thymocyte globulin
RCC	Renal cell carcinoma
RFS	Recurrence-free survival
SIR	Standardized incidence ratio
SMR	Standardized mortality ratio
SOTR	Solid organ transplant recipient

## Appendix A

PubMed

((“Kidney Transplantation” [MeSH Terms] OR kidney transplantation OR renal transplantation OR kidney grafting OR renal grafting) AND (“Colorectal Neoplasms” [MeSH Terms] OR colorectal neoplasm* OR colorectal tumor* OR colorectal carcinoma* OR colon cancer OR rectal cancer) AND (“Epidemiology” [Subheading] OR epidemiology OR incidence OR prevalence OR risk factor* OR “Risk Factors” [MeSH Terms] OR “relative risk” OR “standardized incidence ratio” OR “mortality” OR “survival rate” OR ”mortality” [MeSH Terms] OR “survival rate” [MeSH Terms]))

Web of Science

TS = ((“kidney transplantation” OR “renal transplantation” OR “kidney grafting” OR “renal grafting”) AND (“colorectal neoplasm*” OR “colorectal tumor*” OR “colorectal carcinoma*” OR “colon cancer” OR “rectal cancer”) AND (epidemiology OR incidence OR prevalence OR “risk factor*” OR “risk factors” OR “relative risk” OR “standardized incidence ratio” OR “mortality” OR “survival rate”))

Scopus

TITLE-ABS-KEY((“kidney transplantation” OR “renal transplantation” OR “kidney grafting” OR “renal grafting”) AND (“colorectal neoplasm*” OR “colorectal tumor*” OR “colorectal carcinoma*” OR “colon cancer” OR “rectal cancer”) AND (epidemiology OR incidence OR prevalence OR “risk factor*” OR “risk factors” OR “relative risk” OR “standardized incidence ratio” OR “mortality” OR “survival rate”))

ProQuest

noft((“kidney transplantation” OR “renal transplantation” OR “kidney grafting” OR “renal grafting”)) AND noft((“colorectal neoplasm” OR “colorectal tumor” OR “colorectal carcinoma” OR “colon cancer” OR “rectal cancer”)) AND noft((epidemiology OR incidence OR prevalence OR “risk factor” OR “risk factors” OR “relative risk” OR “standardized incidence ratio” OR “mortality” OR “survival rate”))
